# Assessing the effect of a physical activity intervention in a nursing home ecology: a natural lab approach

**DOI:** 10.1186/1471-2318-14-117

**Published:** 2014-11-18

**Authors:** Carl-Philipp Jansen, Katrin Claßen, Klaus Hauer, Mona Diegelmann, Hans-Werner Wahl

**Affiliations:** Institute of Psychology, Department of Psychological Aging Research, Heidelberg University, Bergheimer Str. 20, 69115 Heidelberg, Germany; Department of Geriatric Research, Agaplesion Bethanien Hospital, Geriatric Center at Heidelberg University, Rohrbacher Str. 149, 69126 Heidelberg, Germany

**Keywords:** Multidimensional physical activity intervention, Nursing home residents and staff, Mixed methods, Automated activity recording, Psychosocial outcomes

## Abstract

**Background:**

Physical activity (PA) is not only an important marker of physical impairment, but also a pathway to improve quality of life and enhance cognitive and social functioning of old individuals. Yet, making interventional use of PA training as a means for prevention and enhancement of quality of life of nursing home residents has found very limited attention worldwide so far. That said, the project ‘Long-term Care in Motion’ (LTCMo) as a part of the INNOVAGE consortium (funded by the European Commission) has the following aims: Overall: Install and assess a socially innovative intervention in the nursing home ecology. Concrete: (a) Conceptualization of a multidimensional intervention program (resident and staff oriented) with the potential to promote PA in nursing home residents; (b) Mixed-methods assessment of the program based on automated recording as well as questionnaire data.

**Methods/Design:**

LTCMo’s PA-related intervention has several components which are applied in parallel manner: (1) Residents are engaged in a physical exercise program that is based on multiple approaches: supervised group sessions, a serious games approach, and specific training in severely impaired persons; (2) Staff members will receive a competence training with a focus on PA motivation and facilitation of residents’ PA engagement. Primary outcome assessment (movement-related behavior of residents) is completely conducted by means of automated data collection strategies (accelerometer-based activity recording, sensor-based life space recording). This is enriched by a broad range of secondary outcomes (e.g., cognitive performance, depression of residents; behavioral and attitudinal components of staff). Pre-, post- and 3-month follow-up assessment will take place in the target intervention setting as well as in a waiting control condition in which we will also replicate the training and its assessment in a later step.

**Discussion:**

Although we are faced with methodological challenges (e.g., rather small sample size; no randomized control trial), we believe that our approach has something to offer and indeed has some unique characteristics that may have the potential to contribute to the enhancement of nursing home residents’ quality of life and at the same time further PA-related research with vulnerable populations at large.

**Trial registration:**

Current Controlled Trials ISRCTN96090441. Registered 31 July 2014.

## Background

Nursing home residents are characterized by old age, high prevalence of multi-morbidity, frailty, mobility impairment, severe cognitive deficits, and depression [1]. In terms of day-to-day behavior, an essential feature of nursing home residents is their very low physical activity (PA), even compared to non-institutionalized older adults in advanced old age [2], although there is also a subgroup depicting excess motor activity and wandering behavior [3]. However, PA is not only an important marker of physical impairment, but also a major pathway to improve quality of life and to enhance motor, cognitive, and social functioning in old age. Empirical evidence supports rather large positive effects of PA on a range of important endpoints such as cardio-vascular fitness, gait and balance, fall reduction, cognitive function, and well-being in the general older population [4, 5]. Moreover, PA training has revealed sizable positive effects in terms of physical and functional ability-related endpoints in those with dementia-related disorders, if efficiently tailored in its application format to the remaining competencies of this specific group [6]. Regarding the nursing home situation, a number of intervention studies, particularly those focusing on intensive muscle training, have shown positive effects in terms of fall reduction [7]. Beyond the fall-related literature, a recent review [8, 9] found only 12 intervention studies—though with mixed design qualities—that are able to speak on the effects of PA training in nursing home residents. Although this scarce empirical platform partially supports the assumption of positive effects of PA on a number of endpoints such as increased motor behavior and activity at large, the overall empirical evidence in this area has remained limited and inconclusive.

At the practical and implementation level, a number of barriers making the exertion of PA difficult for nursing home residents have been identified [10]. Residents’ low physical and cognitive health and functional status including gait and balance problems and sensory impairments likely hinder residents to imagine that a considerable increase in their PA is possible and feasible without taking too many risks. For the same reasons, staff may refuse to think about encouraging residents to be physically active. Indeed, an intervention to increase PA may increase risk exposure and falls, especially in residents with advanced motor impairment [11]. Psychologically, following the classic idea of ‘total institutions’ originally described by Goffman [12], many nursing home residents may feel powerless and low in self-efficacy. In addition, staff may have a tendency for dependency-enhancing behavior regarding residents, thus possibly helping too much and fostering independent behavior too less [13]. All in all, these barriers may result in a vicious circle, in which an overall sedentary life style in the nursing home ecology is reinforced because of a variety of reasons, which may lead to additional impairment in functioning in the longer run via disuse processes and a situation of low engagement at large in everyday life [14].

In research terms, a major difficulty of studies on PA conducted in nursing homes lies in the rigorous assessment of PA. In order to assess changes in PA as the consequence of a respective intervention in a reliable and valid way, subjective measures (e.g., interviews, structured questionnaires) may be hampered due to a decline in cognitive abilities or presence of cognitive impairment like dementia, resulting in recall and response biases. Furthermore, especially nursing home residents tend to be engaged in low intensity activities and PA frequently is of short duration and operating on an irregular basis, i.e., short and slow walking episodes are more the rule than the exception. This situation challenges reliable recall processes, particularly in questionnaire-based research [15, 16].

Therefore objective assessment by high resolution automated activity recording seems mandatory. In addition to quantitative and increasingly also qualitative assessment of PA, ‘life space’ assessment has evolved as an important complementary research perspective. PA assessment includes objective documentation of frequency, duration, and temporal course, of movement characteristics such as lying, sitting, standing and walking. Qualitative data analysis including risk of falling, number, duration, and qualitative features of standing, walking, and transfer actions become increasingly technically feasible. The life space concept, originally questionnaire-based [17], has found its automated recording extension by using sensor installations in the target physical ecology [18], allowing for objective assessment of the physical-spatial context, in which movements take place (e.g., frequency and duration of use of public vs. private locations). The combination of both of these assessment strategies hold promising and complementary research options which have, to our knowledge, not been used in the nursing home ecology so far.

Finally, the kind of PA intervention has to be determined that suits the requirements of the nursing home ecology best, given the range of barriers as described above [19]. Although it seems reasonable to rely on the overall positive evidence and practical experiences gathered with classic PA-enhancing training modules applied to vulnerable older populations such as people with dementia (PWD [6]), additional training components may be advisable. In particular, it may be helpful in motivational as well as stimulus enrichment terms to consider new, creative, and fun-evoking training procedures that may particularly suit nursing home life. For example, training strategies based on a ‘Serious Games’ approach may be a promising addition to focused PA group training programs [20]. Such game-based training typically addresses both motor and cognitive performance. For example, a music-supported stepping task may focus on dynamic postural control, which is mandatory for motor key features such as standing or walking and represents the most effective training approach with respect to fall prevention in older persons [21]. Cognitive training elements of Serious Games-based PA training may relate to cognitive sub-performances such as temporo-spatial orientation, executive functions, timing/reaction time, action in inhibition, attention-related motor cognitive (dual-) tasks, representing important features for motor control and early markers of cognitive decline. Furthermore, given the critical role of staff in the nursing home ecology, it seems helpful to back a direct nursing home residents-oriented PA intervention with staff-based training elements which focus on the significant role of staff as motivational and PA-reinforcing agents [13, 22].

In sum, although PA-related training strategies have considerable potential to enhance quality of life of nursing home residents and potentially also enrich the nursing home as a professional work environment, the respective research literature is very limited. This is where the project ‘Long-term Care in Motion’ (LTCMo) starts from.

### Project aims

LTCMo is part of the INNOVAGE consortium funded by the European commission [23]. In line with the major focus of the European Commission to raise Healthy Life Expectancy (HLE) and overall quality of life of older adults, INNOVAGE aims to showcase a range of social innovations able to contribute to this overarching goal. Within the INNOVAGE architecture, LTCMo addresses the situation of the highly vulnerable older nursing home population and aims to install and assess a respective socially innovative intervention in the nursing home ecology. Driven by the idea of a ‘natural lab’, we are heading for a procedure that comes with innovative and—compared to previous research—significant improvements in terms of its intervention approach as well as its assessment concept. Study aims include:

1. Conceptualization of a multidimensional intervention program operating at different levels of the nursing home ecology with the potential to promote PA behavior in nursing home residents intensively involving residents as well staff members and by these means efficiently counteracting the existing barriers which typically prevent PA exertion.
2. Development of an innovative assessment strategy to comprehensively assess residents’ PA behavior as well as intervention effects, respectively. This assessment concept represents the natural lab component of LTCMo’s social innovation and makes an attempt to unify a practical and hopefully quality of life-enhancing strategy (i.e., the multidimensional intervention) with an ambitious and innovative research and measurement concept in the nursing home setting. The assessment concept as a whole is envisaged to be as reliable, valid, cost-effective, and unobtrusive as possible.
  2.1. Residents’ habitual PA behavior is comprehensively depicted using objective assessment methods, i.e., accelerometer and life space sensor-based data.
  2.2. Assessment of the intervention effects should include pre-, post-, and 3-month follow-up measurement occasions to estimate the short and long-term effects of the intervention in exemplary manner. There is a waiting control condition consisting of a second nursing home ecology, in which at the first stage of assessment no intervention program is conducted to document the natural course without interventional effects.
3. Disseminate findings as intensively as possible based on a guidebook containing a detailed description of the intervention program as well as findings and practical recommendations able to enhance the implementation process of the intervention in nursing home ecologies at large.

## Methods/Design

### Description of intervention

LTCMo’s PA-related intervention has multiple components which are applied in parallel manner: (1) Residents engage in a physical exercise program that is based on multiple approaches: supervised group sessions, a serious games approach, and specific training in severely impaired persons. Its bottom line is a rigorous focus on functional and strength exercises to improve key motor qualifications necessary for mobility, autonomy, and motion security, i.e. standing, walking, sitting down and standing up. It suits as much as possible the needs of the fragile nursing home population; (2) Staff members receive a competence-enhancing training with a focus on PA motivation and facilitation of residents’ PA engagement. To permanently maintain intervention effects, staff is trained to implement training strategies in daily NH routine.

#### Physical exercise training

The exercise intervention of LTCMo relies on long-standing experience and existing evidence of successful PA interventions in old, multimorbid adults with and without cognitive impairment [6, 24, 25] and is at the same time specifically tailored to the needs of the target population of physically and cognitively impaired nursing home residents. The supervised training programs used in this study significantly increased functional and cognitive performance and did not lead to adverse events, neither in long-term use in comparable clinical settings nor in previous intervention studies of the research group [6, 24, 25].

*Supervised group sessions & Specific training in severely impaired persons.* The 45-minute sessions are offered twice a week over a 12-week period in small groups of four to eight residents. Training intensity is increased according to individual progress. To ensure homogeneity, group composition is determined according to residents’ motor and cognitive status, i.e., based on impressions and results derived from performance-based tests and cognitive screening at baseline testing. The exercise sessions are supervised by trained sports scientists to support motivation/adherence and to prevent adverse events such as falls. Trainers are instructed to use communicational strategies developed for use in patients with cognitive impairment. Residents with distinct behavioral problems resulting in disturbance of exercise activities or residents with advanced postural deficits are not included in exercise group sessions. However, they are still eligible for an individual training (Specific training in severely impaired persons) which is based on exercises used in the group training and adapted to the individual abilities of the participants in a one-to-one training situation.

*Serious Games Approach.* In general, exercise training is based on repetitive and standardized training tasks, which guarantee effectiveness, but may fail to attract all participants. The serious games approach provides an alternative mode to motivate these persons to be active with high effectiveness, supported by a “serious”, evidence-based exercise task. The game is constituted as a dual motor/cognitive task. The motor task is based on a progressive functional task (stepping/dynamic postural control) representing the most effective training target for fall prevention [21, 26, 27]. The cognitive task targets different cognitive sub-performances such as divided attention, temporo-spatial orientation, reaction time, and executive performances, which are early markers of cognitive decline [28] and risk factors for falls [26, 27, 29]. For part of such cognitive tasks, trainability has been proven [25].

**Figure 1:**
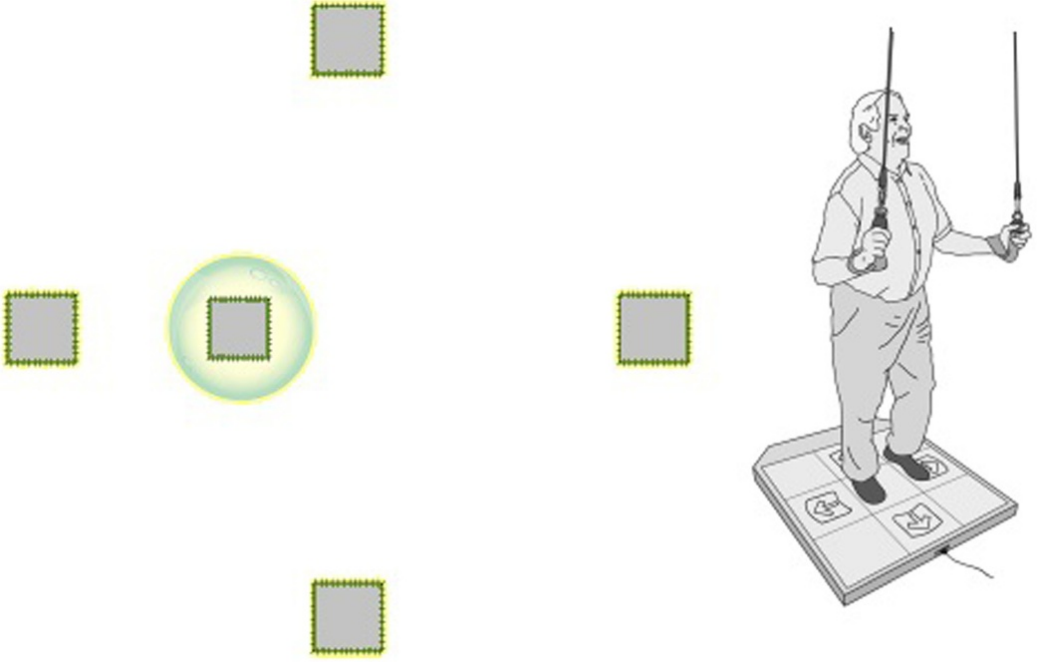
**Screenshot of the game and stepping platform [** [30] **].**

In the current study we use a stepping video game which is based on a modified version of “StepMania dance and rhythm game” [30], see Figure 1. It is constituted as a cognitive-motor training in which the exercise character is substituted by a game character. It will be conducted in small groups of 3-4 residents with only one person playing at a time, supervised by a sports trainer or a trained research assistant. To play the game, the participant has to stand on a dance plate which is connected to a computer via USB. The dance video game screen is projected on a TV screen. A scrolling display of squares moving up, down, right, or left across the screen cues each move and participants have to execute the indicated steps (forward, backward, right, or left) when the squares reach corresponding squares at the top, bottom, right, or left side of the screen (see Figure 1). Participants will have to alternately perform 10 levels of 90 seconds duration each. Difficulty is individually tailored as the program depends on previous individual performance level to prevent overtaxing of users. The standardized program was adjusted to the performance level of frail older adults with and without cognitive impairment in pilot testing prior to the intervention.

#### Physical activity-enhancing competence training for staff

The major aim of the staff centered PA-enhancing competence training is to enable staff members to interact with residents in a way that encourages their PA as much as possible. The conceptual background of the staff training is based on established models of the role of person-environment interactions in long-term care institutions and the significant role of staff [22, 31] as well as on psychological models of health psychology (e.g., Self-Determination Theory [32]); Theory of Planned Behavior [33]; Health Action Process Approach [34], self-regulation theory (e.g., Social Cognitive Theory [35]); model of selective optimization with compensation [36]; dependency-support script [13]); life-span motivational models (e.g., Socioemotional Selectivity Theory [37]); positive messaging [38]). Staff members receive intensive information on the role and evidence related to PA in later life and also regarding the role of negative aging stereotype and concomitant underestimation of residents’ remaining competencies (e.g., [39]). Emphasis is also put on the importance of barriers and facilitators of being physically active and on ways to overcome such barriers in most creative ways. To achieve this goal, staff learns how to use communication and interaction techniques able to encourage residents to be more active (e.g. positivity, motivational interviewing; see also conceptual background above). Based on role play technology and bed-site trainings, staff intervention is also enriched by extensive practice opportunities. In addition, the practical application of communication strategies in caring routines with the goal to develop strategies for upcoming challenges and to monitor the achievement via feedback-loops is a major issue in the later part of the training program and intents to increase the transfer of what has been learned to day-to-day interactions with residents.

The staff training component is served within the framework of the existing regular in-house training schedule and comprises a total of 12 sessions with one session weekly thus amounting to a duration of approximately three months: Eight 1-hour-sessions including theoretical as well as practical contents and four 30-minutes-sessions mainly serving as case discussions. Each session is offered twice a week to facilitate staff attendance due to their revolving shift involvements. An overview of the full program is given in Table 1. Training sessions are planned for a group format of 15 to 20 persons.

**Table 1:** **Contents and sequential flow of the PA-enhancing competence training for staff**

Session	Content
1	Introduction and overview over training program
2	Importance of physical activity in (old) age: Theoretical input and joint discussion
3	The art of behavior change: Theoretical input and joint discussion
4	The role of age stereotypes in caring routines: Theoretical input and joint discussion
5	Communication strategies I: Theoretical input and practical exercises
6	Communication strategies II: Theoretical input and practical exercises
7	Communication strategies III: Practical exercises
8	Feedback on practical application of communication strategies in caring routines and development of respective solutions
9	Case discussion and development of respective solutions
10	Case discussion and development of respective solutions
11	Case discussion and development of respective solutions
12	Case discussion and development of respective solutions

### Research design and sample recruitment

**Figure 2:**
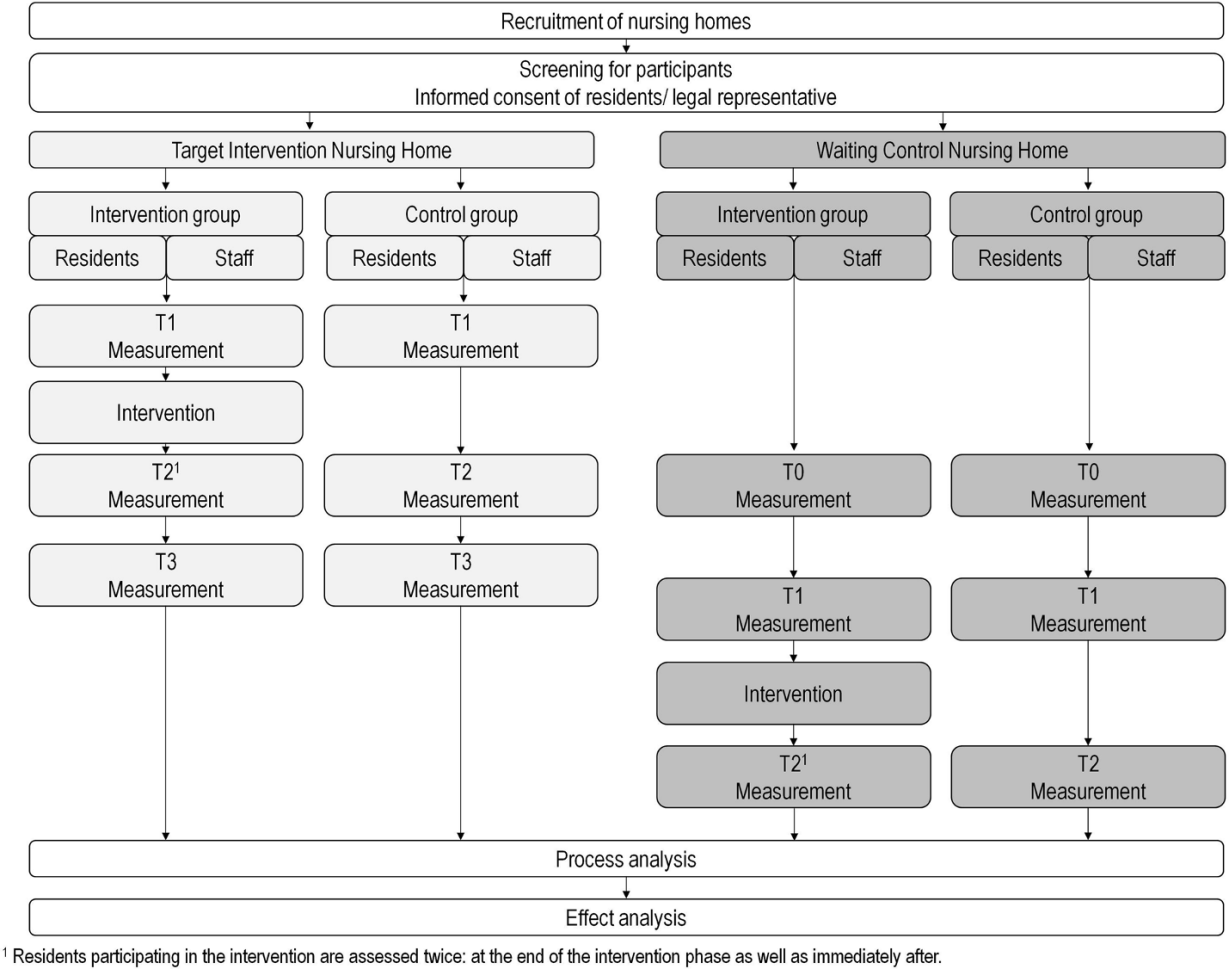
**Study design.**

Figure 2 depicts our research design. We conduct a quasi-experimental pre-post-assessment study with baseline measurement (T1), measurement at the end of the intervention period (T2), and measurement at the end of a 3-month follow-up (T3) in two nursing homes in the Heidelberg area (Germany). In one nursing home a run-in period (T0) will be conducted, documenting status and course without intervention (T0 vs. T1). One nursing home serves as target intervention facility, whereas the other serves as a waiting control condition. Inclusion criteria for residents’ assignment to the intervention are:

1. Permanent resident in included nursing homes
2. Written informed consent (resident/legal representative)
3. For participation in exercise sessions: Ability to stand (supervised group training) or ability to walk (stepping video game). Residents not fulfilling this criterion are still eligible for participation in the assessment procedure.

Exclusion criteria for residents’ intervention participation are:

1. Short-term resident in one of the two nursing homes
2. Behavioral problems resulting in disturbance of exercise activities (with respect to participation in group sessions only).

For residents with severe motor impairment (supervised ability to stand) and challenging behavior, training with one-to-one supervision will be established.

All staff members willing to participate are eligible for inclusion. Residents and staff members not willing to participate in the intervention, but providing informed consent and willing to provide pre- and post-assessment information may be used later as a ‘natural’ control group component in addition to the planned control condition.

As this project has the ambition to assess residents in a ‘natural lab ecology’ of two nursing homes as comprehensively as possible and to include a relative maximum of residents based on our specific approach, we refrain from any formal power calculation. However, we made sure by selecting two homes with more than 100 residents each that complete assessment of at least 50 participants per home will be possible, assuming a drop-out rate of about 50%. Hence, we currently expect about 50 residents in the target intervention setting and about 50 in the waiting control condition. In addition, we expect about 20 staff members to become part of the staff training component and a similar magnitude to serve as controls.

Prior to the onset of the intervention, assessments in terms of self-reports, proxy ratings, performance-based measures, accelerometer, and life space sensor-based data as well as staff data (self-reports only) are conducted (T1). In both facilities, a small group of three staff members is trained to provide the proxy ratings for *all* participating residents (inter-rater reliability will be examined). In the intervention facility, assessments will take place in the final week of the intervention period (PA and life space assessments only) in order to analyze direct effects of the intervention on PA, immediately after the intervention period has been completed (T2), as well as three months after completion of the training (follow-up, T3) by the comprehensive assessment battery. In the waiting control facility, baseline assessment (T0) is followed by a run-in period to document the natural course without intervention. The assessment protocol for the following intervention and post-intervention phase is otherwise identical to the protocol as described above for the first intervention facility.

The process analysis addresses the setting conditions, implementation, and receipt of the intervention as well as its feasibility. Several means of a process analysis are employed to assess the sampling and intervention quality, e.g., characteristics that may cause selective sampling of participants (e.g., residents’ depressive symptoms) are documented as well as the frequency of participation, questionnaire-based evaluations of each staff training session, and supervision of staff’s application of learned techniques. The effect analysis addresses the effectiveness of the intervention components regarding the described outcomes.

### Informed consent and ethical approval

Residents’ relatives, legal representatives, and staff members were intensively informed about the study based on written material as well as oral presentation. To ensure the comprehensibility of the information sheet for residents, two of its versions were already pretested in another nursing home. The version chosen was attested to be comprehensible and of appropriate length. Information events were held for relatives, staff members, and residents. Additionally, staff members were informed during their shift changes. Written informed consent was requested in residents and their legal representatives before the onset of the study (in case those were nominated for persons with severe cognitive impairment).

Ethic approval for the project was obtained from the Ethic Review Board (ERB) of the Faculty of Behavioral and Cultural Studies of Heidelberg University with an approval letter dating from December 19, 2012 (no number or code is provided by the faculty’s ERB)^a^. Two amendments also obtained ethical approval: one addressing the implementation of a sensor system for life space assessment of nursing home residents to monitor activity in publicly accessible areas (letter dating from February 24, 2014), the other addressing the possibility to share our PA data with other data platforms, such as a world-wide documentation of fall events (letter dating from May 28, 2014).

### Measures

All variables identified as primary and secondary outcomes as well as additional variables potentially moderating possible outcomes will be collected from each participant in the intervention condition at the three time points (see Figure 2).

### Outcome measures

A classic assessment challenge in nursing home residents is their highly limited capacity for testing/self-reporting. Therefore, the ambition to make extensive use of automated recording tools of PA also comes—besides its advantages in terms of reliability and validity—with reducing the load of self-reporting. This is further enhanced by referral to proxy ratings, although it is clear that time capacity of professionals in nursing homes is limited and thus proxy ratings must be as short as possible. Besides, major variables that require self-report are assessed in the shortest version possible, without losing quality in terms of reliability and validity in an unacceptable magnitude.

#### Primary outcome measures: residents

*Physical activity assessment*. The primary outcome assessment relies on automated activity and life space recording. Duration and frequency of residents’ *PA* (i.e., lying, sitting, standing, and walking) is recorded using triaxial accelerometers (uSense) fixed at the lower back for 48 consecutive hours. The sensors are non-commercial prototypes, developed by the EU-funded “Farseeing” project (fall detection) and allowing detailed quantitative as well as semi-qualitative data analysis.

*Life space assessment.* Residents’ *life space* is measured objectively using the innovative wireless sensor network method s-net® [40]. Sensors (anchor nodes) are placed in the nursing home and each resident wears a corresponding sensor (end nodes) to track his or her position in time and space continuously. Each battery-operated end node calculates and signals its position at approximately 20 second intervals. The surrounding anchor nodes gather and forward this information to the destination (gateway node connected to the back-end system) according to a communication protocol. This protocol guarantees the network’s energy efficiency and its self-organization, and thus its robustness in the naturalistic setting. Data concerning residents’ covered distance, the duration spent at defined locations and the frequency of changes of locations will be assessed.

Life space assessment parallels PA assessment, i.e. different PA data based on automated recording allows simultaneous recording and combined data analysis.

#### Secondary outcome measures: residents

At the level of performance-based outcome measures, well established clinical tests are conducted. The *Short Physical Performance Battery* (SPPB [41]) is used to assess lower extremity functioning (balance, gait, strength, and endurance). The SPPB requests residents to stand side-by-side, semi-tandem, and full-tandem, for 10 seconds each, to walk a distance of four meters at maximum speed using their walking aid if necessary (two attempts with the better performance being scored), and to perform five timed chair stands with their arms folded across their chest. Each task is scored on a 0-4 scale. Zero points are given if the subject is unable to complete the task. An overall score ranging from 0-12 is created by summation of scores. In addition to the SPPB, the *Timed Up & Go* test [42] is performed. Residents are asked to stand up from a chair, walk three meters (using their walking aid if necessary), return to the chair, and sit down again as fast as they can. With respect to *gait speed,* residents are asked to walk 10 meters at maximum walking speed, using their walking aid if necessary (two attempts with the better performance being scored). The conventional analysis of clinical test data is backed up by a high-tech, qualitative analysis of parameters (DynaPort® Hybrid, McRoberts). The unobtrusive device will be fixed to the residents’ lower back during the test using an elastic belt.

To allow target-specific objective assessment of Serious Game performance and progress, we use the technical data flow of the device for customized assessment. This technically advanced assessment strategy is in line with the high technical standard of assessment used for PA and motor performance in this study, allowing a detailed and comprehensive insight in effects of motor training. In sum, all data assessments regarding PA and physical performance rely on automated recording.

Falls are documented according to a standardized definition [43]. Data on *frequency of falls* is obtained from the nursing homes’ care documentation and standardized questions which participants are asked twice a week. Further, the *Short Falls Efficacy Scale* (Short FES-I [44]) is conducted to assess residents’ concerns about falling. The FES-I has also been validated for use with cognitively impaired persons.

In addition to automated recording and performance-based assessment, we ask staff members to provide a proxy rating on the care and behavior assessment regarding wandering, security measures (restraints, e.g., bed rails, safety belt), frequency of social situations (e.g., visits, staff contact) and activity participation of target residents [45]. Data concerning security measures, falls, and activity participation will be complemented with information from the nursing homes' care documentation.

Residents’ care level will serve as an indicator for their need of assistance with activities of daily living (ADL). It is assessed by the German Health Insurance Medical Service (MDK), varies from 0 *no need of care* (i.e., need for assistance required for less than 90 minutes per day on average) to 3 *in constant need of care* (i.e., need for assistance required around the clock, on average at least for five hours per day). The care level will be taken from the care documentation. Additionally, residents are asked to provide an overall rating on how they perceive their ADL independence based on a single-item approach, i.e. the question “How would you assess—all things considered—your self-dependence?” The answer format is 0 *fully depending on help* to 10 *fully independent*. The item has been proven useful in prior research with older adults as an addition to classic ADL assessment [46].

Concerning the cognitive assessment, the well-established Mini-Mental State Examination (MMSE [32, 47]) will be used. The MMSE is a commonly used and easy to apply screening method and represents a comprehensive screening method including different cognitive sub-performances. In order to also have an external examination of cognitive performance available, MMSE assessments are complemented by the proxy Dementia Screening Scale (DSS) for use by nursing home staff [48]. Staff members rate residents’ memory (e.g., Could he/she remember what happened in the past few days?) and orientation (e.g., Could he/she orient him-/herself in his/her room?) in seven items. The scale proved to be a valid screening tool for proxy use in nursing homes.

We assess *depression* based on the established 15-item *Geriatric Depression Scale* (GDS-15 [49]). The scale has been developed for use in geriatric and vulnerable samples and offers a simple, dichotomous response format; it has also been validated for use with persons with mild-to-moderate cognitive impairment [50–52]. Self-reports on depression will be complemented by a staff-based proxy rating, using the Montgomery-Åsberg Depression Rating Scale (MÅDRS [53]), documented by trained staff members. This task is taken over by trained staff members, i.e., those who know the target residents very well. These raters were endowed with precise explanations of the items’ contents to enhance inter-rater reliability. The scale consists of 10 sevenary items asking for apparent and reported sadness, inner tension, reduced sleep, reduced appetite, concentration difficulties, lassitude, inability to feel, pessimistic, and suicidal thoughts. The MÅDRS has also been validated for use as proxy-assessment for depression of residents in nursing homes [53].

Residents’ satisfaction with life is assessed using a single-item approach, i.e. the question: “How satisfied are you at the moment with your current life?” The answer format is 1 *fully unsatisfied* to 5 *fully satisfied*. The item has been proven useful in prior research with older adults [54].

Furthermore, we assess apathy by use of the *Apathy Evaluation Scale* (AES-D [55–57]) that requires a proxy rating of residents according to 10 statements representing symptoms of apathy (e.g., interest in new experiences, approach to life with intensity, or having initiative).

Finally, we assess how residents perceive their activities as well as their social life. Regarding the former, six items taken from the *Pleasant Events Schedule—Nursing Home Version* (PES-NH [58, 59]) and three additional items in the same format allow residents to provide information on the occurrence as well as on how pleasant they find a given activity. Residents’ perceived social integration is assessed based on the 3-item social loneliness subscale of the established De Jong Gierveld Loneliness Scale [60–62].

#### Potentially moderating variables: residents

Variables that may potentially moderate the training effect are seen on four layers: First, self-efficacy may be important, i.e. residents with higher self-efficacy may profit better from PA training. An economic approach is an ultra-short version of *general self-efficacy* (ASKU [63]), in which residents’ are required to answer only three questions on their general self-efficacy. The scale has been tested psychometrically in several large samples and reference values stratified by age, sex, and education are provided [63]. Second, regarding control beliefs, we use an ultra-short *locus of control scale* (IE-4 [64]); residents are asked two questions on their internal as well as two questions on their external locus of control. The scale has been tested psychometrically in several large samples and reference values stratified by age, sex, and education are provided [64]. Third, we also ask residents to provide their *subjective age*, that is, whether they feel older or younger compared to their calendar age. Subjective age has been found to be significantly related to a number of health outcomes as a recent meta-analysis revealed [65]. Fourth, we make an attempt to assess the overall *motivation to move* based on two single items asking for internal and external motivational forces.

#### Demographic variables and overall health assessment: residents

In terms of demographic variables, we assess residents’ age, sex, marital status, parenthood, years of education, and nursing home entry. For a global health assessment, residents will answer single-item questions on their capacity to move, eyesight, hearing, and whether daily functioning is impaired due to pain. The scale for these questions ranges from 1 *very bad* to 5 *very good* and 1 *extremely* to 5 *not at all*, respectively.

For sample description and use as covariates we document residents’ diagnoses, medication, and BMI using data from the nursing home’s care documentation.

#### Secondary outcome measures: staff

The staff training is meant to impact residents’ PA and additionally to result in a perceived change in professional competence related to PA promotion in residents. To assess this secondary outcome, staff members rate their perceived nursing competency using 18 items from a questionnaire on nursing competence established in Germany (German title: Fragebogen zur pflegerischen Handlungskompetenz [66]). In addition, staff motivation when interacting with residents (e.g., preference for dependence- or independence-supportive behavior, discouraging or encouraging of residents’ activity participation) will be assessed using nine self-developed items, because no established instrument exists in this area. Sample items include “I prefer to have a resident sit in a wheelchair to prevent her/him from falling.”, “I involve residents in ADL as much as possible, even though this takes more time.”, or “I motivate residents to leave their rooms and meet others.” For all assessments, staff members are asked to complete the respective questionnaire at home.

Because not only a change in staff behavior towards residents, but also a possible impact on staff members’ attitudes towards their work is expected due to staff training, the following variables are also assessed: *Work-related consequences of strain* (e.g., difficulty relaxing after work) are measured using the 8-item Irritation Scale [67–69]. *Job satisfaction* will be assessed with a single-item approach, i.e. the question “Overall, how satisfied are you with your job?”. The answer format is 1 *not at all* to 5 *very much*. It is also assumed that staff’s own perceived aging experiences may be touched by the training. Therefore, their *subjective age* (“Some people feel older or younger than they actually are. Apart from your real age, how old do you feel most of the time?”) and their *expectations regarding their own aging* by using the *Expectations Regarding Aging Survey* (ERA-12 [70, 71]) are assessed. The survey includes questions on expectations regarding staff member’s own aging in physical health, mental health, and cognitive function domains. Finally, staff’s age stereotypes in the domains of “friends and acquaintances”, “leisure activities and social or civic commitment”, “personality and way of living”, and “physical and mental fitness” are rated based on the domain-specific age stereotypes questionnaire suggested by Kornadt and Rothermund [72].

#### Demographic variables: staff

At baseline, information on age, sex, marital status, parenthood, and years of education is collected.

## Discussion

Physical training has been established as a major tool for prevention of the occurrence of a range of diseases and loss of functional impairment as well as rehabilitative approaches such as maintaining autonomy, cognitive performance, and the reduction of depressive mood [73]. Although nursing home residents belong to the lower end of the competence continuum in advanced old age, there is no fundamental reason to question such a positive effect in this population. Indeed, one may argue that the nursing home ecology comes with particular advantages in terms of implementing a PA regimen able to improve residents’ quality of life. For example, reachability of target persons for imposing a PA program is rather easy in the “concentrated ecology” of nursing homes, given that the administrative structure has given its commitment to unfold such an intervention. In addition, staff as significant others of residents and well-established, powerful change agents [13] may take over the role of a motivational partner in a highly contingent way, if they receive a respective PA-enhancing training component. That is, the critical part of every PA training in old age (and in other periods of life), i.e. translating an enhanced PA behavior pattern into the turbulences of day-to-day life, may find a particularly suitable, if not ideal platform in the nursing home ecology. Seen in a wider perspective, we believe—as part of the INNOVAGE consortium—that a respective intervention has the potential of a social innovation at large, that is, the shaping of nursing home ecologies toward the better by bringing them more into “motion.”

To achieve this goal, we are currently executing a theory-driven multi-dimensional training program in a nursing home under what we are labeling a natural lab condition. The training follows a multi-dimensional approach and explicitly unifies components of resident-oriented PA training, Serious Games elements, and staff-oriented competence enhancement. As a consequence of our vulnerable target population, primary outcome assessment is completely conducted by means of automated data collection strategies. This strategy is enriched by a broad range of secondary outcomes that rely on proxy ratings, performance-based measures, and self-report data based on answering formats that are simplified as much as possible without questioning the reliable and valid assessment of study variables. In addition, we also assess a range of staff-oriented variables to examine the possible impact of our staff training on staff behavior and attitudes. Going further, we are installing a waiting control condition based on a second nursing home ecology with two intentions: First, the waiting control signifies natural trajectories of outcome measures over time; second, we plan to replicate the training program and do respective pre- and post-assessments after the intervention part has been completed in the target nursing home.

That said, it is obvious that such a design should be seen as a *demonstration study* and comes with a number of challenges and limitations. First, we expect rather small sample sizes in the magnitude of 50 residents and 20 staff members, however, hope to double the intervention-oriented sample sizes in the waiting control group by replication of the training program. On the other hand, we will generate a data space with these small samples that—to our knowledge—currently does not exist worldwide. For example, we will assess different dimensions of resident motion in space and time based on automated recording and performance-based variables. Indeed, we regard our assessment as a rather unique combination of detailed quantitative and semi-qualitative PA data in combination with life space data which are not available so far to our knowledge for this population. Envisaged secondary data analysis will also be possible, such as the relation among depression and activity in nursing home residents.

Second, considerable missing data at the various levels of assessment is expected and we are also facing the challenge of potentially invalid self-report data. Here, we hope that such missing data can be “compensated” to a major extent by using different data layers. For example, self-report data obviously will not play the key role in our resident population and will be “compensated” by the automated recording efforts. Third, it is clear that we are not following the strict criteria of a RCT format. That is why we talk about a demonstration study research design that will of course need replication based on a stronger design at a later point in time, if emerging results are promising. Fourth, we purposefully are not intending to separate the possible differential effects of the various intervention components. Fifth, we see the data-analytic challenges coming with our demonstration study design. At first glance, it seems obvious that we have quite an imbalance between data density and number of variables and sample size. However, intensive data collection based on intensively observed smaller sample sizes is emerging in many areas of behavioral and health research in aging and beyond and many innovative statistical procedures have been suggested to optimally treat such a data situation [74, 75].

In sum, although we clearly see the challenges ahead, we believe that our approach has something to offer and indeed has some unique characteristics that may have the potential to contribute to the enhancement of nursing home residents’ quality of life and at the same time further PA-related research with vulnerable populations at large.

## Endnote

^a^In Germany, ERBs are not always assigning numbers to their decisions. This is also true in our case.

## Abbreviations

ADL: Activities of Daily Living
ERB: Ethic Review Board
HLE: healthy life expectancy
LTC: long-term care
LTCMo: Long-term Care in Motion
MÅDRS: Montgomery-Åsberg Depression Rating Scale
MMSE: Mini-Mental State Examination
PA: Physical activity.
